# Screening for Mild Cognitive Impairment with Speech Interaction Based on Virtual Reality and Wearable Devices

**DOI:** 10.3390/brainsci13081222

**Published:** 2023-08-21

**Authors:** Ruixuan Wu, Aoyu Li, Chen Xue, Jiali Chai, Yan Qiang, Juanjuan Zhao, Long Wang

**Affiliations:** 1 College of Computer Science and Technology (College of Data Science), Taiyuan University of Technology, Taiyuan 030024, China; 2 College of Information, Jinzhong College of Information, Jinzhong 030600, China

**Keywords:** mild cognitive impairment, virtual reality, wearable EEG, language analysis, cognitive assessment, classification

## Abstract

Significant advances in sensor technology and virtual reality (VR) offer new possibilities for early and effective detection of mild cognitive impairment (MCI), and this wealth of data can improve the early detection and monitoring of patients. In this study, we proposed a non-invasive and effective MCI detection protocol based on electroencephalogram (EEG), speech, and digitized cognitive parameters. The EEG data, speech data, and digitized cognitive parameters of 86 participants (44 MCI patients and 42 healthy individuals) were monitored using a wearable EEG device and a VR device during the resting state and task (the VR-based language task we designed). Regarding the features selected under different modality combinations for all language tasks, we performed leave-one-out cross-validation for them using four different classifiers. We then compared the classification performance under multimodal data fusion using features from a single language task, features from all tasks, and using a weighted voting strategy, respectively. The experimental results showed that the collaborative screening of multimodal data yielded the highest classification performance compared to single-modal features. Among them, the SVM classifier using the RBF kernel obtained the best classification results with an accuracy of 87%. The overall classification performance was further improved using a weighted voting strategy with an accuracy of 89.8%, indicating that our proposed method can tap into the cognitive changes of MCI patients. The MCI detection scheme based on EEG, speech, and digital cognitive parameters proposed in this study provides a new direction and support for effective MCI detection, and suggests that VR and wearable devices will be a promising direction for easy-to-perform and effective MCI detection, offering new possibilities for the exploration of VR technology in the field of language cognition.

## 1. Introduction

Mild cognitive impairment (MCI), a common neurodegenerative disorder, leads to memory, logic, language, and executive dysfunction. It also accelerates cognitive decline, and severe cognitive impairment can create barriers to social independence and interaction [1]. MCI, a precursor to Alzheimer’s disease (AD) [2], is also often considered a transitional stage between cognitive decline in healthy aging and dementia, with a 1-year conversion rate from MCI to dementia of 18.4% [3]. Studies have shown that the number of people living with dementia doubles every 20 years, and by 2050, the number of people living with dementia worldwide will increase from the current 50 million to 139 million, imposing a heavy burden on both society and families [4]. Although AD is one of the most common forms of dementia, there are no disease-modifying treatments for AD [5], and it is an irreversible disease. Fortunately, however, neuropathological changes associated with cognitive impairment emerge a decade or more before the onset of overt symptoms of AD [6], making the diagnosis of MCI in the early stages of AD an important challenge.

One reason why the diagnosis of MCI is often delayed is that most patients confuse cognitive decline in healthy aging with cognitive impairment due to MCI and do not undergo clinical examination at an early stage. Studies have shown that 76% of patients fail to recognize the onset of cognitive impairment at an early stage [7]. In addition, the current diagnosis of MCI is mainly clinical, but the diversity of assessment criteria and assessment processes worldwide has led to inconsistent diagnoses of MCI, as well as etiological investigations [8]. Diverse diagnostic criteria and diagnostic guidelines have led to diagnostic delays [9].

Currently, paper-and-pencil screening tests (e.g., the mini-mental state examination (MMSE) and the Montreal cognitive assessment (MoCA)) are commonly used for cognitive testing. Although the utility of these tests is high with a good reliability and validity [10], these tests need to be administered under the guidance and supervision of a professional instructor, thus limiting their widespread use in daily life. Certain neuroimaging tools (e.g., magnetic resonance imaging, etc.) and biomarkers can provide accurate diagnostic results, but their wide application is limited by their high cost and invasiveness [11]. Therefore, exploring effective diagnostic options for MCI remains a focus of the biomedical field and the field of neurology.

Biomarkers play a key role in the diagnosis of MCI as an important change in patients with MCI. In the ongoing exploration of biomarkers, non-invasive mobile wearable devices have gradually become the focus of attention. Xie et al. [12] studied the walk speed, stride length, stride time, stride frequency, and stride time variability in patients with amnestic mild cognitive impairment (aMCI) using a sensor-based wearable gait measurement device, and found that walk speed and stride length would decrease and stride time variability would increase in aMCI patients. This demonstrated that gait impairment in daily walking can be used as an indicator for the detection of cognitive dysfunction and validated the potential of sensor-based wearable gait measurement devices to be used as a tool for screening for cognitive impairment. Similarly, Mulas et al. [13] studied gait speed, stride length, stride frequency, and double support duration in older adults using wearable inertial sensors (IMUs) and found that patients with cognitive impairment exhibited a low speed, low stride length, low cadence, and high double support duration. This demonstrated the correlation between mobility and cognitive impairment and the validity of wearable inertial sensors (IMUs) applied to the assessment of cognitive impairment in the elderly. Borhani et al. [14] analyzed memory performance, brain oscillations at rest, and interchannel amplitude squared at rest in 43 older adults using wearable and wireless electroencephalogram (EEG) headphones to measure key physiological brain signals during rest and working memory tasks. They found that alpha and beta oscillations at the right parietal sites were greater with a lower memory extraction accuracy, while a better working memory performance was significantly associated with increased power in the left parietal delta and theta bands. Alharbi et al. [15] used CorSense to compare the indicators of heart rate variability (HRV) in the MCI group with the healthy control (HC) group at rest and found that the HRV indicators were lower in the MCI group. This suggested that HRV could be used as a biomarker to identify MCI. Iliadou et al. [16] used the MindWave Mobile EEG Headset to study the differences in brain activity between older adults with MCI and those with subjective cognitive decline (SCD) in the virtual supermarket test (VST). They found that at the completion of the VST, the beta and theta band power increased in the MCI group compared to the SCD group. In addition, in the resting state, the alpha, beta, delta, and theta band power increased in the MCI group compared to the SCD group. It was also verified that the performance parameters of VST combined with the EEG signal are more beneficial for the detection of cognitive impairment. Geng et al. [17] proposed a sleep EEG-based MCI detection method that extracted sleep slow waves and spindles features from 40 participants and obtained a 93.46% classification accuracy in a gate recurrent unit (GRU) network. This demonstrated that sleep EEG signals are more accurate than waking EEG signals in MCI detection, and again proved that sleep slow waves and spindles features can be used as early biomarkers of AD. Thus, the physiological data from the sensors offer new possibilities for the early and effective clinical detection of MCI.

In addition, clinical studies have shown that speech and language changes in AD patients may appear in the first few years of clinical symptoms. Balogh et al. [18] investigated temporal parameters in the MCI and HC groups in a semantic fluency task and showed that three task-independent parameters, the number of silent pauses, the average length of silent pauses, and the average word transition time, were task-independent parameters which were more useful for MCI detection. In addition, it was verified that they had similar categorization abilities to the traditional fluency measurement parameters. Vincze et al. [19] investigated participants’ temporal speech parameters in the immediate recall of the film description task, previous day task, and delayed recall of the film description task. Their study showed that speech rate, number and length of pauses, pause frequency, and signal could successfully distinguish mild Alzheimer’s disease from healthy individuals, and they also found that the higher the severity of dementia, the higher the percentage of the total speech duration accounted for by pauses. Wang et al. [20] studied the lexical, semantic, syntactic, phonological fluency, and acoustic characteristics of 75 participants in picture descriptions and spontaneous self-presentations. The study demonstrated that MCI was significantly characterized by a decrease in speech production, accompanied by signs of increased disfluency and a linear trend of decreasing semantic content and syntactic complexity. This language change produced by MCI patients provides ample opportunities for the efficient detection of MCI and related research.

However, most of the current studies have been limited to tapping the relevance of biomarkers or speech features to MCI patients, and few studies have explored the effect of these features in MCI classification. Furthermore, no research team has applied the combination of EEG signals and speech signals, as well as time digitization parameters, to aid in the diagnosis of MCI. Therefore, this paper proposed a MCI detection scheme based on EEG signals, speech signals, and time digitization parameters, which incorporates physiological signals, speech signals, and digital cognitive parameters. A more accurate, objective, and efficient MCI classification framework was established for the automatic tracking and assessment of cognitive impairment in older adults. The language task of the study used the scene description task based on the fully immersive virtual reality (VR) developed by our team. VR is able to collect more ecological indicators of cognitive function, including those that cannot be measured by classical traditional assessment tools, while providing real-time visual, tactile, and auditory stimuli. These provide assistance in assessing different levels of cognitive function. In addition, cognitive testing in a VR environment may increase patient interest and engagement in the diagnostic process. In order to stimulate participants’ language functions to a greater extent, this study combined VR technology and the field of language cognition, breaking through the limitations of traditional cognitive testing modalities (e.g., paper tests, tablet tests, etc.) and providing a highly ecological and immersive testing environment for cognitive assessment from a more novel perspective. To our knowledge, this is a rare study that combines VR technology with MCI language diagnosis, thus offering new possibilities for the exploration of VR technology in the field of language cognition. Meanwhile, this study used a wearable EEG device, MUSE 2, to record participants’ EEG signals during the task. Unlike previous studies that were limited to EEG signals in the resting state, this study explored the brain activity of MCI patients and healthy individuals under language stimulation in a non-invasive and low-cost manner and investigated the sensitivity of EEG to detect abnormal brain changes associated with MCI. In addition, this study broke the limitation of previous studies that only investigated the relevance of linguistic features or physical features to MCI patients by extracting MCI linguistic features and EEG features and exploring their role in MCI detection. The proposed MCI detection scheme based on EEG signals, speech signals, and time digitization parameters can achieve the effective classification of MCI patients, while providing further support for the application of VR technology and wearable devices in the field of cognitive impairment, improving the popularity and daily possibility of cognitive impairment assessment methods.

The main contributions of this study are as follows. Firstly, an efficient, accurate, and objective MCI classification algorithm based on EEG signals, speech signals, and time digitization parameters was proposed. The algorithm can effectively distinguish MCI patients from healthy individuals and fill the gap of insufficient research in the field of combining physiological manifestations, linguistic manifestations, and digitized parameters to assist in MCI diagnosis. Secondly, studies have suggested that EEG signals, speech signals, and digitized parameters show good synergistic effects in MCI diagnosis. The multimodal data provided a more comprehensive and adequate analysis compared to single-modal as well as dual-modal features, which provides practical support for the fact that multimodal data can have a good synergistic effect on MCI detection. In addition, it was confirmed that the voting strategy can improve the classification accuracy under multitasking. Thirdly, this study explored the contribution of language features and EEG features to the classification of MCI patients and healthy individuals, bridging the gap in distinguishing MCI patients from healthy older adults in terms of linguistic differences and physiological signal differences. Finally, this study provides new ideas for the application of VR technology in the field of MCI language cognition, as well as validation and support for the application of wearable devices in the field of neuropathology.

## 2. Materials and Methods

**Figure 1:**
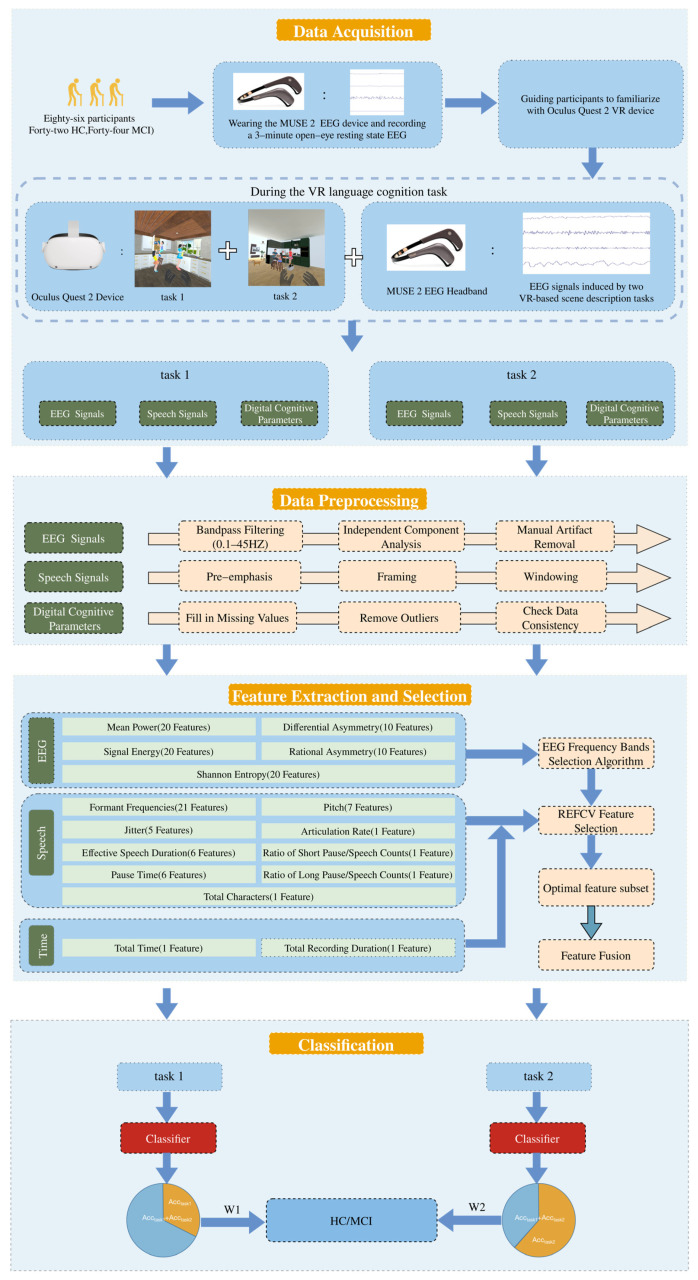
The framework of the proposed MCI detection scheme based on EEG data, speech data, and digital cognitive parameters.

The steps of the proposed MCI detection scheme based on EEG, speech, and digital cognitive parameters, including data acquisition, data preprocessing, feature extraction and selection, and MCI classification are shown in Figure 1. Each step is elaborated in detail in the following subsections.

### 2.1. Data Acquisition

#### 2.1.1. Participants

In this study, 86 participants were recruited and divided into MCI and HC groups. The MoCA scale and the MMSE scale are widely used cognitive screening scales, whereas the MoCA is more potent for the detection of early cognitive dysfunction than the MMSE [21]. The MMSE is a good discriminator between healthy older adults and dementia, and it is of good value in screening for dementia but of limited value in identifying healthy older adults with MCI. Therefore, all participants in this study had a score ≥ 24 on the MMSE, excluding participants with significant cognitive impairment [21]. Kandiah et al. [22] showed that MoCA is a reliable tool for predicting early cognitive decline, and MoCA scores ≤ 26 significantly increased the risk of progressive cognitive decline. According to this criterion, 44 cases (23 males/21 females) who scored ≤26 on the MoCA test were included in the MCI group, and the remaining 42 cases (19 males/23 females) were included in the HC group. In addition, participants met the following criteria: (1) age 65 years or older; (2) no visual or hearing impairment; (3) no mental illness or cerebral stroke due to cognitive dysfunction; (4) able to use the VR device normally; (5) participants did not have any lifestyle that affected EEG signals (e.g., did not consume any caffeine products in the day prior to conducting the experiment, as brain activity is disturbed by caffeine [23]). All participants signed an informed consent form after the researchers explained the purpose, procedure, risks, and benefits of the study.

We statistically analyzed the clinical and demographic characteristics of the participants in both groups. A normality test was first performed, and all characteristics of the participants in each group satisfied normality. We performed independent samples *t*-tests for continuous variables such as age, educated years, MoCA scores, and MMSE scores (*t*’-test was used for characteristics with heterogeneity of variance), whereas for categorical variables such as sex, we used a chi-square test. Table 1 summarizes the clinical and demographic information of the 86 participants. It can be seen that the MCI and HC groups were matched in terms of age, sex, and education level, while there were significant differences in terms of MoCA scores and MMSE scores (*p*-values all less than 0.001).

#### 2.1.2. Equipment

**Figure 2:**
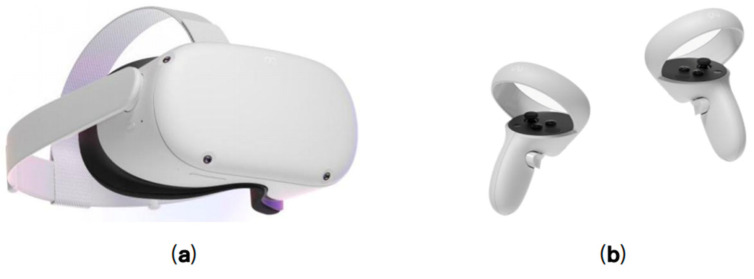
Oculus Quest 2. (**a**) Head-mounted display. (**b**) Two controllers for Oculus Quest 2.

In this study, VR equipment and EEG equipment were mainly used, and the equipment is described as follows:

The VR device used in this study was the Oculus Quest 2, consisting of a head-mounted display (HMD) and two Oculus touch controllers (Figure 2). It allows for more realistic and naturalistic interactions, enabling participants to interact immersively through the Oculus touch controllers. Bailey et al. [24] found that the HMD of Oculus Quest had a high usability and low discomfort, providing further support for the feasibility of applying Oculus Quest to VR research. The Oculus Quest 2 has been widely used in various studies for a wireless all-in-one VR device that is low-cost, portable, and has a high motion tracking accuracy. Craig et al. [25] evaluated the reliability of Oculus Quest applied to balance detection, and found that changes in postural control due to different types of visual field manipulation could be effectively detected. This suggested that the low-cost Oculus Quest offers new prospects for balance training. Carnevale et al. [26] applied Oculus Quest in a shoulder rehabilitation application and demonstrated its high accuracy in upper extremity motion accuracy measurements, further supporting the reliability and promise of Oculus Quest as a VR tool for patient rehabilitation. Munoz et al. [27] designed an immersive VR exercise based on Oculus Quest for patients with dementia, providing evidence for using VR technology to provide a more tailored and healthy lifestyle to patients with dementia. These studies all confirmed the validity and reliability of Oculus Quest and demonstrated that VR technology may be a future trend for patients with cognitive impairment and even further disorders.

For the EEG device, a low-cost, wirelessly portable, non-invasive visual wearable EEG device (MUSE 2) was used in this study to collect EEG signals from participants. MUSE 2 breaks through the time-consuming, labor-intensive, and costly limitations of traditional EEG recording methods, and it is widely available while being easy to use. Ratti et al. [28] demonstrated that MUSE can be used in research by comparing the quantitative EEG signals and retest reliability of medical grade and consumer EEG systems, showing that higher quality EEG data can be collected with the consumer EEG system MUSE. With the gradual development of the MUSE headset, more and more research teams are adopting MUSE as a device for EEG signal acquisition for related studies. Krigolson et al. [29] used MUSE to explore the relationship between perceived cognitive fatigue and human event-related potential (ERP) and electroencephalography (EEG) oscillations in a large sample, while validating that portable EEG devices can be a viable research tool to effectively measure relevant changes in the brain. In addition, there are also research teams that have applied MUSE to stroke disease detection [30], heat sensation measurement [31], engagement classification [32], and mood detection [33]. All of these studies have validated the MUSE wearable device as a reliable and effective EEG data collection device by exploring the changes in biomarkers extracted from EEG data. Pu et al. [34] validated the feasibility of using MUSE to measure pain in long-term care residents with dementia, suggesting that EEG signals may be a potential biomarker for pain measurement in patients with dementia, providing further support for the study of MUSE as an adjunctive assessment tool for cognitive impairment.

**Figure 3:**
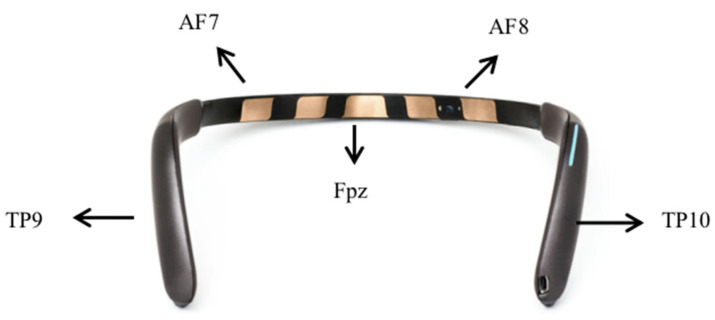
Muse 2 EEG headband for measuring the activity of the brain via four electrodes: TP9, AF7, AF8, and TP10.

The MUSE 2 EEG headband is a four-channel EEG device, consisting of four dry electrode channels (TP9, AF7, AF8, and TP10) and reference electrodes (Fpz). AF7 and AF8 are the two forehead electrodes, while TP9 and TP10 are two ear electrodes. The electrode positions of the Muse 2 EEG headband are shown in Figure 3. Brain activity data measured by MUSE 2 are transferred to Mind Monitor software (version number 2.3.0) at a sampling rate of 256 HZ via a Bluetooth serial connection stream for offline analysis.

#### 2.1.3. Experimental Procedure

**Figure 4:**
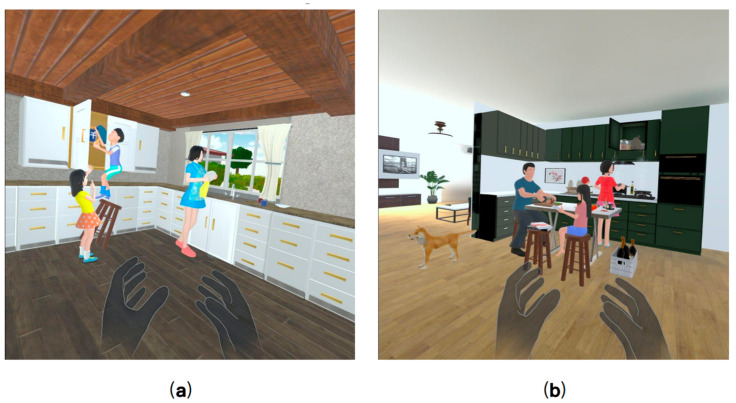
Two VR-based scene description task interface diagrams. (**a**) Task 1; (**b**) Task 2.

First, the researcher explained the purpose of the study and the entire process of the experiment to the participants. After obtaining their informed consent, the participants were asked to complete the MMSE and MoCA scales, according to which the participants were divided into the MCI group and the HC group. After a short break, participants sat in a comfortable chair in a relaxed position and were asked to wear the MUSE 2 EEG device and record a 3-min open-eye resting state EEG. Since the Oculus Quest 2 VR device was unfamiliar to most of the participants, the participants were allowed to observe the device in a hand-held manner before wearing it. Immediately afterward, the Oculus Quest 2 VR device was worn by the participant and the participant was guided to experience the virtual environment for 5 min. During the guided session, participants were allowed to ask the researcher any questions they had. After completing the familiarization phase, participants were asked to complete two speech tasks that we developed and deployed via the Oculus Quest 2 VR device (shown in Figure 4). Specifically, each speech task provides a full range of visual and auditory experiences, allowing users to observe a range of elements of the scene and describe the observed scene in as much detail as possible. During this period they can explore each VR scene in all directions, including user walking and item grabbing. There is no limit to the amount of time the user can explore and describe. Participants’ speech data and digital cognitive parameters during the task were obtained through the VR device, and EEG data during the task were obtained through the MUSE EEG device.

### 2.2. Data Preprocessing

Due to the continuous change with time, the EEG signal is usually unstable [35], and the EEG signal can be affected by various factors and produce different noise. Therefore, preprocessing was performed first to eliminate artifacts. The collected EEG data were preprocessed using the EEGLAB toolbox, which is a widely used open-source software environment for processing EEG data. First, a bandpass filter of 0.1–45 HZ was applied to clean up the EEG signal by filtering the data to reduce filtering artifacts such as direct current shifts. Secondly, the independent component analysis (ICA) method was used to address the problem of possible artifact interference in the collected EEG data such as blink, ECG, and EMG. Harpale et al. [36] showed the important role of ICA for EEG data processing through their study and also showed that ICA plays an important role in the EEG signal detection of diseases. Finally, the shape of the signal was observed to manually eliminate data with artifacts or noise that had not been eliminated before.

The preprocessing steps of speech data in this experiment include pre-emphasis, framing, and windowing. The Librosa library for Python and other libraries were mainly used to complete these steps. Firstly, the signal spectrum was flattened and the resonance amplitudes were made close to each other by pre-emphasis. Secondly, the continuous speech signal was divided into a series of 10–30 ms long frames, which helped to extract the useful features of the speech signal. Finally, windowing was performed to eliminate the signal discontinuities that might be caused at the ends of each frame, and the Hamming window was used in this study.

Digital cognitive parameters based on the VR device recorded participants’ cognitive performance during the completion of language tasks, and the use of these data as digital biomarkers for the classification of MCI individuals is also one of the issues explored in this study. Therefore, before extracting the features, the raw data of digital cognitive parameters need to be subjected to data cleaning, including filling in missing values using mean values, removing outliers, and checking data consistency.

### 2.3. Feature Extraction and Selection

For the EEG data recorded for each VR language cognition task, the use of frequency-domain metrics (e.g., frequency band energy) is a widely used feature extraction method in AD signal classification [37,38] and also for other EEG-based disease classification. Four types of frequency domain features were extracted from all channels of the EEG signal, including mean power (MP), signal energy (SE), spectral entropy, and asymmetric features. The frequency bands were divided into δ (1–4 Hz), θ (4–8 Hz), α (8–13 Hz), β (13–30 Hz), and γ (30–45 Hz). The mean power was calculated for each frequency band on each channel for a total of 20 features, i.e., the five bands of the four channels of the MUSE headband. The signal energy is obtained by calculating the sum of squares of the absolute values of all samples of the EEG signal for a total of 20 features, i.e., the five bands of the four channels of the MUSE headband. The spectral entropy was defined as the Shannon entropy (ShE) of the signal power spectrum; the entropy measures the predictability of random variables and can be used as a measure of signal complexity. The total number of features obtained from the Shannon entropy was 20, including the 5 bands of the four channels of the MUSE headband. The asymmetric features include differential asymmetry (DASM) and rational asymmetry (RASM). DASM is the difference in band power between the left and right hemispheres corresponding to the electrode positions (TP9 and TP10, and AF7 and AF8). There are 10 DASM features, including 5 band powers for the two electrode pairs used. RASM is the band power ratio of the left and right hemispheres corresponding to the electrode locations (TP9 and TP10, and AF7 and AF8), and a total of 10 RASM features were used, including 5 band powers used by the two electrode pairs. In this study, the above 80 EEG features were analyzed separately for each VR language task.

For the speech data recorded by each VR language cognition task, this study extracted 2 types of speech features, including acoustic features and rhythmic features, using the Python-based audio processing libraries Librosa and Signal_Analysis2. Acoustic features include formant frequencies (F1, F2, F3) and jitter (local, local absolute, rap, ppq5, ddp). Formant frequencies are a major feature of the voice, and the accurate detection of formant frequencies is useful for distinguishing different rhymes and for improving the recognition of the semantics of speech. Jitter is the measurement of random perturbations in period length, a measure of fundamental frequency and amplitude perimeter variation. A total of five different types of jitter were calculated for the acoustic intervals. The rhythmic features include pitch, pause time, and effective speech duration. The minimum, maximum, range, mean, standard deviation, skewness, and kurtosis were calculated for the formant frequencies and pitch. The minimum, maximum, range, mean, sum, and standard deviation were counted for pause time and effective speech duration. In addition, we counted the ratio of long pause/speech counts, ratio of short pause/speech counts, total characters, and articulation rate. Thus, a total of 49 speech features were analyzed for each VR language task. For the digitization parameters during each VR language cognitive task, the total time spent on the test and the duration of the recording were extracted for each participant.

In order to maximize the classification accuracy of the extracted features, this study utilized the band selection algorithm proposed by Arsalan et al. [39] for EEG features. The band selection algorithm works to select optimal bands by maximizing the classification accuracy achieved from all combinations of the five bands. Afterwards, the wrapper feature selection method was applied to the EEG features at the optimal frequency band, speech features, and digitization parameters to select the optimal subset of features. This study used recursive feature elimination with cross-validation (RFECV), which uses cross-validation based on recursive feature elimination (RFE) to retain the best-performing features. Unlike RFE, which eliminates features by feature importance, this method uses cross-validation for different feature combinations to obtain the importance of different features to the score and finally retains the best feature combinations. Task 1 retained 4 EEG features, 3 speech features, and 1 digital cognitive parameter, while Task 2 retained 5 EEG features, 4 speech features, and 1 digital cognitive parameter. The EEG features, speech features, and digital cognitive parameters selected for each test were fused into a feature vector for the final MCI classification detection. The final feature vectors obtained for the two VR-based language cognition tasks were 8 and 10 in length, respectively.

### 2.4. Classification Algorithm

In this study, four different machine learning algorithms were used to classify the extracted and selected feature vectors for MCI, including decision tree (DT), random forest (RF), support vector machine (SVM), and XGBoost. DT is based on a tree structure that divides the samples according to the impurity of the nodes and progressively constructs a predictive model of the attribute structure. RF is an integrated learning algorithm with DT as the base evaluator, which improves the accuracy and stability of prediction by constructing multiple decision trees, and combines the prediction results of multiple decision trees into the final result by voting in accordance with the principle of minority rule. In this study, the tree-count of RF was set to 150. The SVM algorithm maps the feature vector of an instance to some points in space, and by solving the separated hyperplane that can correctly partition the training data set and has the largest geometric separation, the features close to the hyperplane will be classified into their respective classes, which is one of the supervised learning algorithms. In this study, the kernel function type of the SVM was set to the radial basis function (RBF). XGBoost is an integrated learning algorithm based on the gradient boosting algorithm (GBDT). As a stepwise forward additive model, using the idea of boosting to reduce the risk of overfitting by adding the loss function of the regular term, the XGBoost algorithm does not use the search method but directly uses the first-order derivative and second-order derivative values of the loss function, and greatly improves the performance of the algorithm by pre-ranking, weighted quantile, and other techniques. In this study, the hyperparameter gamma was set to 0, the learning rate (eta) was set to 0.3, and n_estimators was set to 120.

Different cognitive tasks have different degrees of contribution to the MCI detection results, so a weighted voting strategy was used in this study. The features selected under the three modalities for each cognitive task were fused to form the final feature vector, and the classification result obtained from training the classifier would be multiplied by a weight as the classification result for this cognitive task. Finally, the weighted results corresponding to various categories in all cognitive tasks were summed up separately, and the category corresponding to the largest value was the final MCI classification result. The weight value was determined by the classification accuracy of each cognitive task. Specifically, the weight of the classification accuracy of each cognitive task to the sum of the classification accuracies of all cognitive tasks was used as the decision weight of this cognitive task to reflect the influence of different cognitive tasks on the MCI classification performance.

## 3. Experimental Results

### 3.1. Performance Analysis of Band Selection and Feature Selection

**Figure 5:**
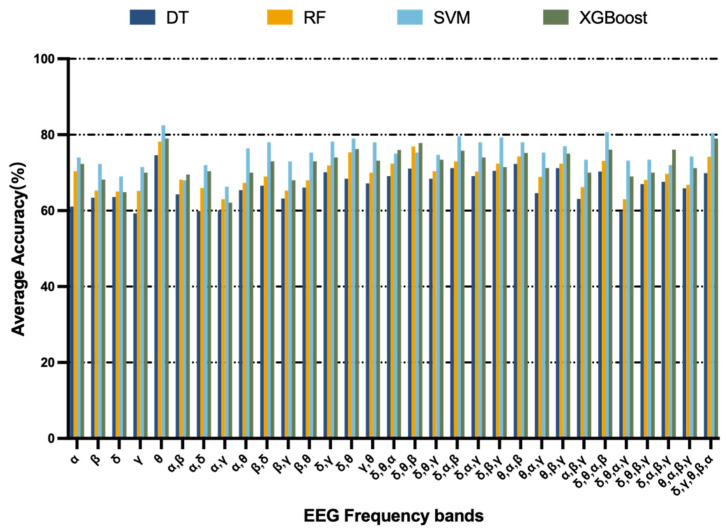
Classification accuracy of various classifiers based on features extracted from different combinations of EEG frequency bands.

In order to identify the bands that discriminate MCI patients at maximum validity, this study used a band selection algorithm [39] to select the most significant EEG bands that correctly classify MCI to the maximum extent. In the band selection algorithm, a leave-one-out cross-validation scheme was used, where the number of folds was equal to the number of instances in the experiment. The training of the data was formed on all but one instance, and the iterations were repeated 1000 times to calculate the average accuracy of all folds to select optimal EEG bands. Figure 5 shows the average accuracy of performing optimal band selection for the EEG data in this study by using the band selection algorithm. It can be observed that the SVM classifier gave the highest accuracy in the theta band. The results showed that the highest MCI classification accuracy was achieved for all band combinations when applying the features of the theta band. Thereafter, feature selection was performed in the theta band using RFECV. The feature subsets selected for Task 1 including MP (TP9), ShE (AF8), SE (AF7), and DASM (TP9 and TP10). For Task 2 including MP (TP10), ShE (TP9), SE (AF7), DASM (TP9 and TP10), and RASM (AF7 and AF8). For the speech data, Task 1 applied RFECV to generate a subset of features including the sum of effective speech duration, ratio of short pause/speech counts, and total characters. The subset of features selected for Task 2 including jitter, sum of effective speech duration, ratio of short pause/speech counts, and total characters. For the digital cognitive parameters, they both chose the feature of total time spent on the test.

### 3.2. Classification Performance Analysis

To explore the classification performance of features under different modalities in VR language cognition tasks, we compared the classification performance of all VR language cognition tasks under four machine learning classifiers (DT, RF, SVM, XGBoost) for EEG features, speech features, time digitization parameters features, and different fused features. The classification performance was measured based on four metrics (accuracy, precision, recall, and F1 score). Leave-one-out cross-validation was used to verify the validity of the proposed method in the study. In order to reduce the influence of randomness in feature selection and classification model construction, leave-one-out cross-validation was repeated 100 times in this study, and the average value was taken as the final classification result.

The results are shown in Table 2 and Table 3, indicating that the MCI detection accuracy differs under different modalities. The best classification accuracy was achieved with the fused features of the three modalities, where the highest classification accuracy (87%) was obtained under the SVM classifier, and the best results were obtained for precision, recall, and F1 score (87%, 86%, and 87%, respectively) (Table 3). In addition, the SVM classifier showed the best classification performance in different modalities. When using data from only one modality as features, the EEG features achieved a higher classification performance compared to the time digitization parameters features and the speech features (Table 2). We also compared the classification of features fused under both modalities. The results showed that when fused features of EEG and speech were used, a higher classification performance was achieved, second only to the classification performance under the fused features of the three modalities (Table 3). Overall, a higher classification performance was obtained using the fused features of both modalities than under a single modality.

In addition, we investigated the effect of different linguistic tasks on MCI classification when using multimodal data and the effect of weighted voting strategies on classification performance. As shown in Table 4, the results showed that the weighted voting strategy had a significant impact on improving the MCI classification performance, and the use of SVM with the RBF kernel produced the highest detection accuracy of 89.8%. In addition, the fused features using all language tasks had a higher performance than the classification using only a single task, second only to the classification performance under the weighted voting strategy.

The above comparison experiments show that the fused features of multimodal data can provide a greater contribution to MCI detection compared to single-modal or bimodal data, while adding a weighted voting strategy for different VR language cognition task can further improve the classification problem in multitask situations. The experimental results demonstrate the effectiveness of the MCI detection scheme proposed in this paper for differentiating MCI patients from healthy individuals.

### 3.3. Language Performance of MCI Patients and Healthy Controls

**Figure 6:**
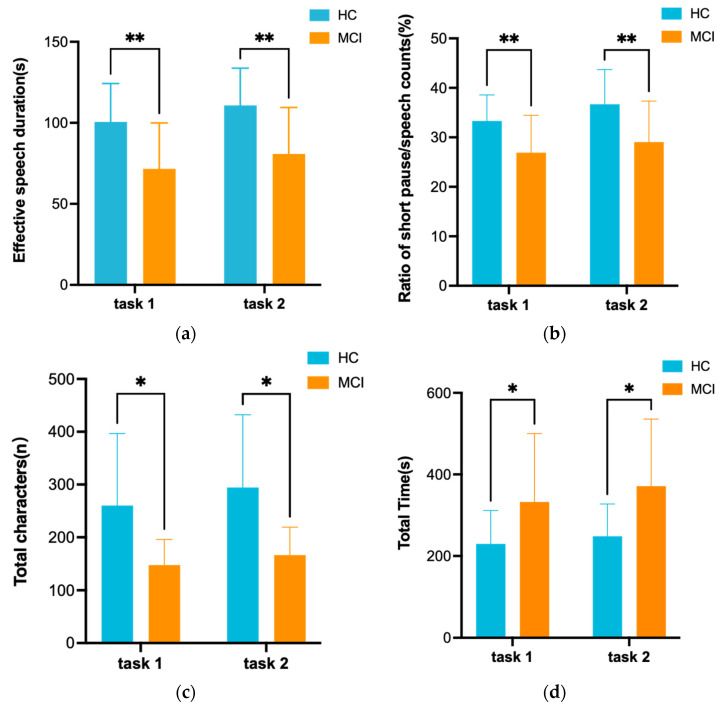
The *t*-test was used to statistically analyze the speech performance of the MCI patients and the HC group in the two VR-based scene description tasks. (**a**) Effective speech duration; (**b**) ratio of short pause/speech counts; (**c**) total characters; (**d**) total time spent on the test. In the figure, * indicates *p* < 0.05, ** indicates *p* < 0.01.

A statistical analysis of some of the linguistic characteristics of the investigated groups was performed, and the results are shown in Figure 6. The bar graphs show the mean, variance, and their differences between groups of the linguistic characteristics data for MCI patients and healthy controls. MCI groups typically exhibit shorter periods of effective speech than healthy controls, and this change in function may be due to the reduced ability of MCI patients in lexical extraction, syntactic processing, and discourse planning, a change in the temporal domain that indicates specific impairments in the cognitive language domain of MCI patients. During the description, we found that the ratio of short pause/speech counts was lower in MCI patients. Short pauses usually represent pauses within sentences and long pauses represent pauses between sentences. This indicated that a higher use of complex sentences was observed in healthy individuals, whereas the opposite was the case for MCI patients. The participants’ speech fluency was measured based on the number of filled pauses within the discourse and the number of speech counts, and the appearance of this reduced speech fluency in MCI patients may be due to a decrease in executive function, as lexical retrieval places a higher demand on the controlled process after the exhaustion of automatically activated words [40,41]. Compared to healthy controls, MCI patients produced significantly fewer characters in their discourse, which may be a result of the reduced cognitive ability of MCI patients leading to a significant modification of the features describing the number of characters. Fleming et al. [42] explain the reduction in discourse content as “a deficiency in the executive skill associated with semantic processing, which is responsible for retrieving, maintaining, monitoring, and manipulating semantic representations.” Meanwhile, the reduction in the number of characters produced further leads to a reduction in effective speech duration. As for the total time spent on the test, we found that the total time spent by MCI patients was longer. We observed that they would spend more time exploring the VR environment, which may have contributed to the fact that MCI patients took longer to complete the test. Furthermore, as expected, we observed that MCI patients lost details of the scene during description and they preferred to use a large number of conjunctions, and the heavy use of conjunctions indicated hesitation to evaluate the scene further by repeating conjunctions to gain time.

### 3.4. Physiological Signal Performance of MCI Patients and Healthy Controls

**Figure 7:**
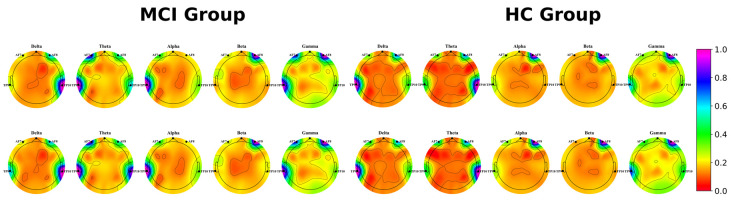
Visualization of brain activity in terms of power spectral density in different EEG bands for both groups of participants during the two phases of the experiment. Row 1: resting state; Row 2: during the cognitive task.

We visualized the power spectral density of EEG data from MCI patients and healthy controls at rest, as well as during the task, as shown in Figure 7. The color bar on the right side of the figure represents the depth of color, where purple represents high brain activation and red represents low brain activation. From the experiments, it can be concluded that: (1) The two groups produced significantly different brain activity in the theta and delta bands. (2) With language stimulation, an increase in power spectral density at different levels was detected in MCI patients and healthy individuals at lower frequency bands compared to the resting state, and a more pronounced increase in power spectral density was detected at lower frequencies in the MCI group compared to healthy individuals. In addition, we found that the theta band had a more pronounced different brain activity visually compared to the delta band.

## 4. Discussion

This study combined VR-based language tasks and EEG to propose a multimodal MCI detection scheme based on language signals and EEG signals, as well as digitization parameters, while using decision-level fusion to represent the contribution of different VR language cognitive tasks to MCI detection. This contributes to the early detection of MCI while collecting valuable information related to cognitive–behavioral and physiological data to analyze the changes in MCI patients from a more comprehensive perspective. To our knowledge, this is a rare study using a VR language cognition application and wearable sensors for assessment, as well as monitoring language and physiological changes in MCI patients, providing a valuable reference for the development of more effective MCI detection protocols and preventive measures in the future. Specifically, we fused the features selected from the three modalities of speech signals, EEG signals, and digitization parameters for each VR language cognitive task, while using a weighted voting strategy to achieve fusion at the decision level using differences in weights. Regarding the cognitive assessment results, an accuracy of 89.8% was achieved, which is better than the methods proposed by Shahzad et al. [43] and Toth et al. [44] in studies distinguishing MCI from HC. It not only showed that the fusion of speech signals, sensor physiological signals, and digital cognitive parameters has good synergistic effects in screening MCI patients, but also showed that decision-level fusion can improve the accuracy of MCI detection under multiple cognitive tasks. As we expected, the fusion of multimodal data can provide more comprehensive information on the physiological differences between MCI patients and healthy individuals, and dig deeper into the cognitive changes of MCI patients. This study combined speech signals, EEG signals, and time cognitive parameters for the first time, providing support for a new direction in the application of multimodal data fusion for the detection of neurocognitive disorders. In addition, the superiority of decision-level fusion for multitask classification was demonstrated, and a weighted voting strategy was used to reflect the differences in MCI detection by different VR cognitive tasks, which can further improve the MCI classification accuracy.

Second, regarding cognitive tasks, we focused on the language cognitive domain. Clinical studies have shown that both speech and language changes are associated with AD pathology and the progression of these changes is related to the severity of the disease [45]. We observed that differences in language performance between the MCI group and healthy control group were also significantly different in terms of diagnosis. Language is closely associated with a wide range of cognitive functions, including working memory, attention, and executive control, and has continuous interaction with different areas of the brain. Compared to healthy controls, the MCI group had a worse language performance on the task. As described in Section 3.3, the MCI group had a reduced ability to produce discourse, as well as a lack of verbal fluency and verbal validity. This phenomenon arises due to the fact that the successful completion of language tasks requires a large number of cognitive operations, as well as linguistic knowledge and processing integrity, again validating the possibility that language decline may have occurred in the prodromal phase of the disease. In addition, statistical analysis revealed that the total time spent by participants in completing the VR language cognitive task performed well on the MCI test, which we explain as a possible reason for the difference between the two groups due to the fact that MCI patients need to spend a longer time exploring in the VR environment compared to healthy individuals. In other words, compared to paper-based and tablet-based methods of MCI detection, VR-based language applications can capture cognitive parameters that are not captured by traditional methods, enabling a more comprehensive and in-depth study of cognitive changes in MCI. It also validated that the MCI detection protocol proposed in this study can reveal the earliest points at which cognitive interventions are most useful, which also provided further support for the application of VR and wearable physiological devices in the field of neurocognitive disorders.

Furthermore, this study analyzed the changes in brain activity of MCI patients and found that the characteristics of their EEG theta band provided a greater contribution to MCI classification, in agreement with Musaeus et al. [46] who showed that power changes in the theta band are an early marker of cognitive decline in dementia due to AD. We recorded EEG data from both groups of participants and visualized their power spectral densities. The experimental results showed that during the scene description, MCI patients detected a more pronounced increase in power spectral density at lower frequencies, and theta had a visually more pronounced difference in brain activity. Meanwhile, under language stimulation, MCI patients and healthy individuals produced different levels of activation of brain activity in the low frequency band compared to the resting state, suggesting that these VR language cognitive tasks can cause the activation of relevant brain regions in participants. Furthermore, brain activity was activated to a greater extent in MCI patients with language stimulation compared to healthy individuals, and possible reasons for this could include the fact that people with cognitive decline require more brain activity to complete cognitive tests.

However, there are still other limitations of this study that need to be considered in future research. First, the limited sample size of this study inevitably limits us to a relatively simple classifier model structure. The sample size should be expanded in the future to provide the conditions to accurately evaluate the classification performance of different classifiers. Second, only two population groups of subjects were considered in this study: MCI and HC, while different stages of cognitive impairment (e.g., mild dementia, severe dementia, or different types of dementia, etc.) were not considered separately. Further inclusion of different stages and types of cognitive impairment in future analyses will further validate the extensiveness of using the MCI detection protocol proposed in this study. Finally, the multimodal MCI detection scheme proposed in this study was limited to the language cognitive domain. In the next study, we will explore whether the multimodal MCI detection scheme can be used as an efficient classification algorithm under other cognitive domains (e.g., executive function, memory, attention, etc.) to more comprehensively study the cognitive parameters and physiological signals of participants under different cognitive domains.

## 5. Conclusions

This study proposed a multimodal MCI detection scheme based on EEG and speech signals, as well as digital cognitive parameters, using the VR device and the wearable EEG device MUSE 2 to detect MCI through the designed VR-based language task. The collected EEG data were selected using a band selection algorithm to choose the optimal band; the RFECV method was applied to select a subset of features for EEG features under the optimal band, speech signals, and digital cognitive parameters. Regarding the features selected under different modality combinations for all language tasks, we performed leave-one-out cross-validation for them using four different classifiers. We then compared the classification performance under multimodal data fusion using features from a single language task, features from all tasks, and using a weighted voting strategy, respectively. The experimental results showed that the highest classification accuracy of 87% was achieved by the RBF kernel SVM classifier under multimodal data fusion. This indicated that the feature classification effect under the fusion strategy was better than that of the features from a single data source, providing new practical support for VR devices and wearable EEG devices to produce good synergy for MCI detection. Meanwhile, the overall classification performance was further improved with an accuracy of 89.8% after using the weighted voting strategy, which proved the effectiveness of the MCI detection scheme proposed in this study. In addition, we analyzed the differences exhibited in the physiological signals and speech signals of the two groups of participants. With the development of sensor technology and the wide application of VR technology, the multimodal MCI detection framework proposed in this study provides a new direction and support for effective MCI detection. In a broader sense, this study provides novel ideas for the combined application of VR and wearable devices in the field of MCI language cognition detection.

## Figures and Tables

**Table 1:** Clinical and demographic characteristics.

	HC (*n* = 42)	MCI (*n* = 44)	*p*-Value	Difference	95%CI
Age avg (std)	67.71 (4.947)	68.81 (6.172)	0.531	−1.101	−4.609, 2.408
Sex (F[%]/M[%])	23[55]/19[45]	21[48]/23[52]	0.393	/	/
Educated years avg (std)	10.18 (1.629)	9.71 (1.637)	0.349	0.467	−0.526, 1.460
MoCA avg (std)	27.24 (0.752)	21.71 (2.312)	<0.001	5.526	4.360, 6.692
MMSE avg (std)	27.53 (1.625)	25.32 (0.702)	<0.001	2.207	1.531, 2.883

**Table 2:** Performance comparison of classification frameworks with different classifiers under individual modality features from VR devices and wearable EEG devices.

Classifier	EEG				Time Digitization Parameters				Speech			
	Acc	Pre	Rec	F1	Acc	Pre	Rec	F1	Acc	Pre	Rec	F1
DT	0.74	0.72	0.76	0.74	0.71	0.75	0.69	0.72	0.73	0.72	0.75	0.73
RF	0.76	0.75	0.74	0.75	0.73	0.77	0.74	0.76	0.74	0.76	0.72	0.74
SVM	**0.83**	**0.81**	**0.80**	**0.81**	0.79	0.80	0.78	0.79	0.81	0.82	0.79	0.80
XGBoost	0.78	0.79	0.77	0.78	0.75	0.73	0.75	0.74	0.76	0.75	0.91	0.82

**Table 3:** Performance comparison of classification frameworks with different classifiers under multimodal features of VR devices and wearable EEG devices.

Classifier	EEG + Time Digitization Parameters				EEG + Speech				Time Digitization Parameters + Speech				EEG + Speech + Time Digitization Parameters			
	Acc	Pre	Rec	F1	Acc	Pre	Rec	F1	Acc	Pre	Rec	F1	Acc	Pre	Rec	F1
DT	0.74	0.73	0.78	0.76	0.78	0.75	0.76	0.75	0.73	0.75	0.74	0.74	0.82	0.84	0.80	0.82
RF	0.77	0.76	0.76	0.76	0.79	0.77	0.78	0.78	0.77	0.73	0.76	0.75	0.83	0.84	0.81	0.83
SVM	0.83	0.83	0.82	0.82	**0.84**	**0.84**	**0.84**	**0.84**	0.83	0.81	0.81	0.81	**0.87**	**0.87**	**0.86**	**0.87**
XGBoost	0.78	0.79	0.79	0.79	0.80	0.79	0.80	0.79	0.78	0.79	0.78	0.79	0.85	0.85	0.86	0.86

**Table 4:** Classification performance under multimodality using features of the single language task, features of all tasks, and using the weighted voting strategy.

Task	Classifier	Acc	Pre	Rec	F1
task 1	SVM	0.826	0.849	0.818	0.833
task 2	XGBoost	0.841	0.820	0.874	0.846
all tasks	SVM	0.874	0.870	0.864	0.867
weighted voting	SVM	**0.898**	**0.895**	**0.883**	**0.889**

## Data Availability

The data presented in this study are available on request from the corresponding author. The data are not publicly available due to the nature of this study, the participants in this study did not consent to the public sharing of their data, and the data at the individual level contain potentially sensitive information, introducing ethical constraints.
